# MicroRNA-Mediated Suppression of Oncolytic Adenovirus Replication in Human Liver

**DOI:** 10.1371/journal.pone.0054506

**Published:** 2013-01-22

**Authors:** Erkko Ylösmäki, Sergio Lavilla-Alonso, Sari Jäämaa, Markus Vähä-Koskela, Taija af Hällström, Akseli Hemminki, Johanna Arola, Heikki Mäkisalo, Kalle Saksela

**Affiliations:** 1 Department of Virology, Haartman Institute, University of Helsinki and HUSLAB, Helsinki University Central Hospital, Helsinki, Finland; 2 Molecular Cancer Biology Program, Biomedicum Helsinki, University of Helsinki, Helsinki, Finland; 3 Cancer Gene Therapy Group, Molecular Cancer Biology Program and Transplantation Laboratory, University of Helsinki, Helsinki; 4 Institute for Molecular Medicine Finland (FIMM), University of Helsinki, Helsinki, Finland; 5 Department of Pathology, Haartman Institute, University of Helsinki and HUSLAB, Helsinki University Central Hospital, Helsinki, Finland; 6 Department of Gastrointestinal Surgery, Helsinki University Central Hospital, Helsinki, Finland; National Institute of Health, United States of America

## Abstract

MicroRNAs (miRNAs) are important and ubiquitous regulators of gene expression that can suppress their target genes by translational inhibition as well as mRNA destruction. Cell type-specific miRNA expression patterns have been successfully exploited for targeting the expression of experimental and therapeutic gene constructs, for example to reduce pathogenic effects of cancer virotherapy in normal tissues. In order to avoid liver damage associated with systemic or intrahepatic delivery of oncolytic adenoviruses we have introduced the concept of suppressing adenovirus replication in hepatic cells by inserting target elements for the liver-specific miR122 into the viral genome. Here we show using *ex vivo* cultured tissue specimens that six perfectly complementary miR122 target sites in the 3′ untranslated region of the viral E1A gene are sufficient in the absence of any other genetic modifications to prevent productive replication of serotype 5 adenovirus (Ad5) in normal human liver. This modification did not compromise the replicative capacity of the modified virus in cancer tissue derived from a colon carcinoma liver metastasis or its oncolytic potency in a human lung cancer xenograft mouse model. Unlike wild-type Ad5, the modified virus did not result in increased serum levels of liver enzymes in infected mice. These results provide a strong preclinical proof of concept for the use of miR122 target sites for reducing the risk of liver damage caused by oncolytic adenoviruses, and suggest that ectopic miR122 target elements should be considered as an additional safety measure included in any therapeutic virus or viral vector posing potential hazard to the liver.

## Introduction

MicroRNAs (miRNAs) are small non-coding RNA molecules 20–24 bp in length that negatively regulate gene expression through binding to complementary sequences typically residing within the 3′ un-translated region (UTR) of mRNAs. Partial sequence complementarity between miRNA and the target mRNA leads to repression of mRNA translation, whereas a high degree of sequence complementarity can guide destruction of the target mRNA [1]. Already more than 1000 human miRNA precursor sequences have been deposited in miRBase [2], and more than 50% of cellular mRNAs have been estimated to be under miRNA regulation [3], [4]. miRNAs are expressed in tissue- and differentiation state-specific patterns, and are often differentially expressed or deleted in various human cancers [5], [6], [7].

Several viruses infecting humans have been shown to contain target sequences for human miRNAs that can suppress viral gene expression [8], [9], but the role of this phenomenon as an antiviral mechanism remains controversial [10]. Nevertheless, numerous recent studies have shown that by introducing artificial miRNA target elements into viral genomes the miRNA machinery can be experimentally exploited to modify the replicative tropism of both RNA and DNA viruses (reviewed by Kelly and Russell [11]. By preventing replication in specific tissues accounting for viral pathogenicity it is possible to generate live attenuated vaccine viruses, as well as safer oncolytic viruses for the treatment of cancer. For example, Barnes at al. used a miRNA targeting strategy to control poliovirus tissue tropism for developing rationally attenuated polio vaccines [12]. By inserting target sites for muscle-specific miRNAs into the viral genome Kelly et al. were able to overcome the lethal myositis occurring in tumour bearing mice infected with Coxsackie A21 virus without compromising the oncolytic potential of this virus [13].

Adenoviruses have been actively studied as tools for cancer virotherapy [14], [15], [16]. Several approaches have been used to increase tumour selectivity of oncolytic adenoviruses. Chemical or genetic modifications of capsid proteins have been made to augment infection of cancer cells [17]. Replacement of the E1A promoter with tumour- or tissue-specific promoters has been used to target adenovirus replication into tumours, such as prostate carcinoma [18]. In addition, removal of the E1B55K reading frame as well as specific deletions within E1A have been used to increase tumour selectivity of adenovirus replication [19].

Systemic administration of adenoviruses leads to infection of hepatocytes, which can cause severe liver toxicity [20], [21]. To improve the safety of oncolytic adenoviruses we have introduced a miRNA-based approach for engineering adenoviruses that are suppressed in their replication by the liver-specific miR122 [22]. By inserting miR122 target elements in the 3′UTR of E1A gene we could strongly reduce E1A expression in cells of hepatic origin. Subsequently, Cawood at al. used the same strategy to show reduced E1A protein levels in murine liver *in vivo* as well as in cultured human hepatocytes *in vitro* [23], [24]. However, in order to achieve potent liver-specific suppression of adenovirus replication additional E1A expression-reducing modifications have been necessary [22], [25].

Thus, it has remained unclear if miRNA-based engineering alone has the potential to prevent liver toxicity associated with systemic administration of oncolytic adenoviruses. In this study we show in normal human liver tissue strong suppression of otherwise unmodified adenovirus 5 carrying six copies of miR122 target elements in E1A 3′ UTR. These results provide a definitive validation for introducing miR122 targets into oncolytic adenovirus constructs as a safeguard of the liver.

## Results

### Construction and Characterization of a Novel miRNA-targeted Adenovirus

**Figure 1:**
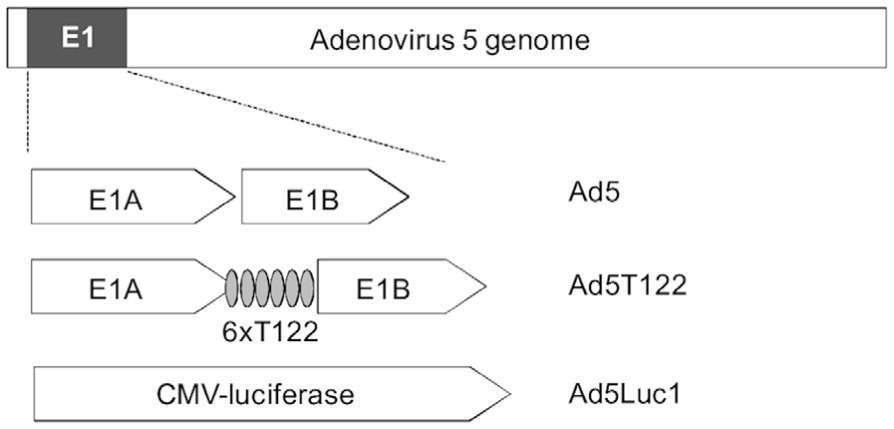
Schematic illustration of the virus constructs used in this study. Ad5 is a wild-type serotype 5 adenovirus containing an unmodified E1 region. In Ad5T122, six copies of miR122 target elements were introduced in the 3′UTR of E1A gene. Ad5Luc1 is a replication-deficient virus in which the whole E1-region has been replaced with a CMV-driven firefly luciferase gene.

To generate a miRNA-targeted version of wild-type adenovirus 5 (Ad5 in Fig. 1), we inserted six copies of target elements with perfect sequence complementarity for the liver-specific microRNA miR122 in the 3′UTR of the E1A gene (Ad5T122 in Fig. 1). As a control virus, we used a non-replicative virus containing the firefly luciferase gene under the control of the cytomegalovirus (CMV) promoter in the E1-region [26] (Ad5Luc1 in Fig. 1).

**Figure 2:**
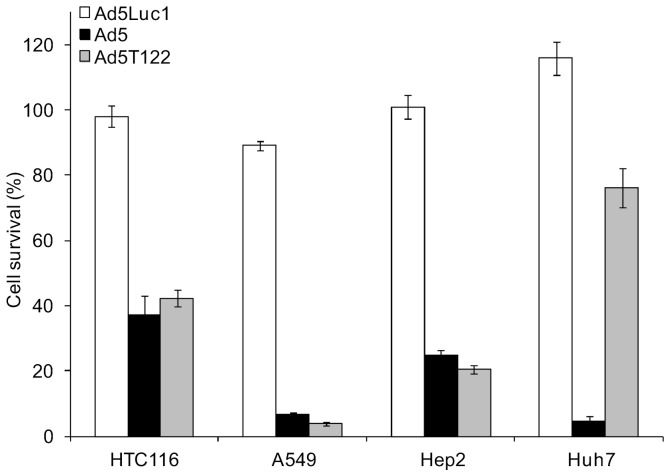
Comparison of cell killing by Ad5 and Ad5T122 in a panel of cancer cell lines. Four cell lines of non-hepatic (HCT116, A549, and Hep-2) or hepatic (Huh7) origin were infected with Ad5Luc1 (non-replicative virus control), Ad5, or Ad5T122 at an MOI of 0.05. Cell survival in the infected cells was measured 7 days (Hep-2 and A549) or 9 days (HCT116 and Huh7) post-infection using an ATP-based cell viability assay, and plotted on the y-axis as the percentage of the control values measured from uninfected cultures. The data are presented as the mean of six repetitions ± standard error.

Huh7 hepatocellular carcinoma cells resemble normal hepatocytes in that they express significant amounts of the liver-specific miR122 [27], and have previously been used as an in vitro model for adenovirus infection of liver cells to demonstrate the capacity of miR122 target sites to down-regulate E1A expression [22], [23]. Thus, we infected Huh7 cells together with a panel of cancer cell lines of non-hepatic origin with Ad5, Ad5T122 or Ad5Luc1 at a multiplicity of infection 0.05. Because of the non-hepatic origin of the latter cell lines they were not expected to express miR122. To confirm this, we quantified the functional miR122 expression in these cell lines using a previously validated dual luciferase assay [22]. As expected, a strong miR122 target element-dependent suppression of reporter gene expression was observed in Huh7 cells, whereas no evidence for miR122 expression in A549, HCT116, or Hep-2 cells could be detected (Figure S1). The extent of lytic cell killing caused by the spread of the infection in this panel of cell lines was monitored using a colorimetric cell viability assay (Figure 2). The E1-deleted, non-replicative Ad5Luc1 did not have a significant effect on the viability of any of the cell lines tested, whereas infection with Ad5 led to destruction of all cell lines with a variable but high efficiency. Very similar cytopathicity was observed following Ad5T122 infection in all of the non-hepatic cell lines. By contrast, cell death caused by Ad5T122 was strongly reduced in Huh7 cells, indicating that replication of Ad5T122 could be attenuated by miR122.

To confirm that the attenuation of Ad5T122 in Huh7 cells was indeed specifically due to silencing by miR122 we generated stable cell lines in which the critical miRNA machinery component Argonaute 2 (Ago2) had been targeted for silencing with lentivirally transduced anti-Ago2 shRNAs. We also examined the effect of miR122 inhibition by a transfected antagomir designed against miR122. In both cases the suppressive effect of the miR122 target elements on replication and cytopathicity of Ad5T122 in Huh7 cells was almost completely abolished (Figure S2).

### Ad5T122 is Strongly Attenuated in Normal Human Liver but Replicates Well in Tumour Tissue

**Figure 3:**
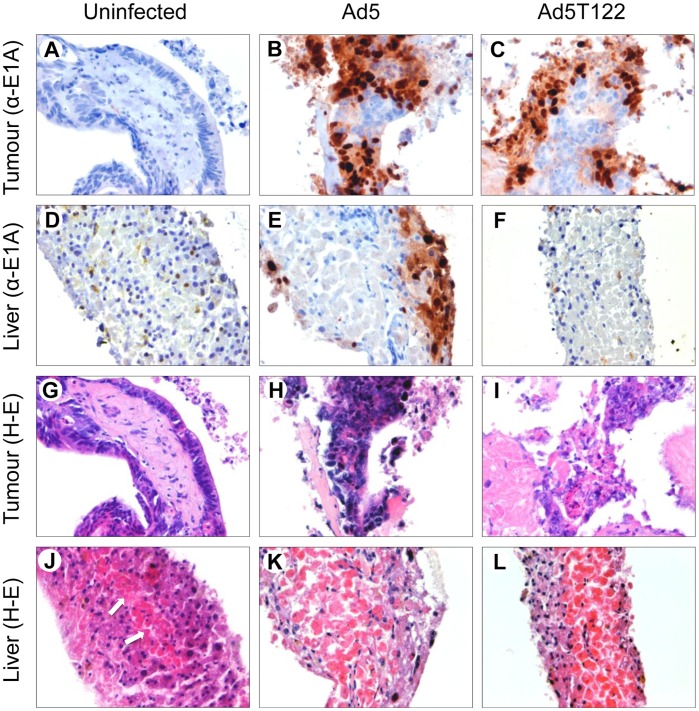
Immunohistochemical analysis of Ad5 and Ad5T122 infection in ex vivo tissue cultures of normal human liver and colorectal carcinoma liver metastasis. Precision-cut tumour (top and third row of panels) and liver (second row and bottom panels) tissue cultures were left uninfected (left panels) or infected with 10^7^ PFU (in 2 ml of media) of Ad5 (middle panels) or Ad5T122 (right panels), and fixed five days later for staining with haematoxylin-eosin (panels G-L) or an antibody against the viral E1A protein (panels A–F). Necrosis developing in the central area of the uninfected control tissue (panel J) is indicated with arrows.

To examine the potential of the miR122-mediated suppression in controlling Ad5T122 replication in human liver we turned into an experimental system based on *ex vivo* culturing of precision-cut human liver tissue slices [28], [29]. Since human Ad5 is normally only capable of an abortive genome replication in murine cells [30], we considered this *ex vivo* human liver infection model as a superior study system for preclinical evaluation of adenoviral-induced liver toxicity. Indeed, this system allowed us to follow productive adenovirus infection in the presence of all the cell types within the context of the normal three-dimensional architecture of the human liver (see Figure 3).

The same *ex vivo* experimental system was also used to confirm that the robust replication of Ad5T122 observed in cancer line lines (Figure 2) could be recapitulated in human tumour tissue. To this end, we compared the replicative capacity of Ad5 and Ad5T122 in precision-cut tumour slices obtained from a colorectal carcinoma liver metastasis, which is a particularly relevant malignancy for this study. Oncolytic adenoviruses have already been evaluated in clinical trials for treatment of patients with colorectal cancer liver metastases [31], [32], and the value of hepatocyte detargeting of the virotherapy is especially evident in this instance.

The histomorphological evaluation based on haematoxylin and eosin staining of the uninfected precision-cut liver slices showed that although towards the end of the 5 day follow-up some increase in the amount of well demarcated coagulation necrosis was observed in the central areas of the slices, the outer cell layers remained viable for the entire duration of the experiments (Panel J in Fig. 3). In the precision-cut tumour slices the integrity of the histology was preserved even better, and the majority of the cells appeared viable after five days of *ex vivo* culture (Panel G in Fig. 3).

Immunohistochemical analysis of the Ad5-infected tissue slices with an antibody against adenovirus E1A protein showed considerable increase in signal intensity in the liver as well as in the tumour as the infection proceeded (Figure 3, panels B and E, and data not shown), indicating efficient viral replication and spread in both tissues. A very similar staining pattern was observed when immunohistochemistry was used to follow the spread of Ad5T122 in the tumour tissue (Figure 3, panel C). This confirmed that inclusion of the miR122 target sites had not compromised the replicative potential of Ad5T122 in the tumour tissue, as we had already observed in cancer cell lines (Figure 2). By contrast, immunostaining of the Ad5T122-infected liver tissue did not reveal any specific signal for E1A (Figure 3, panel F), and appeared identical to the analysis of the uninfected control liver tissue (Figure 3, panel D). Thus, we concluded that miR122 in normal human liver tissue could exert a powerful negative regulation on the target site-containing virus.

**Figure 4:**
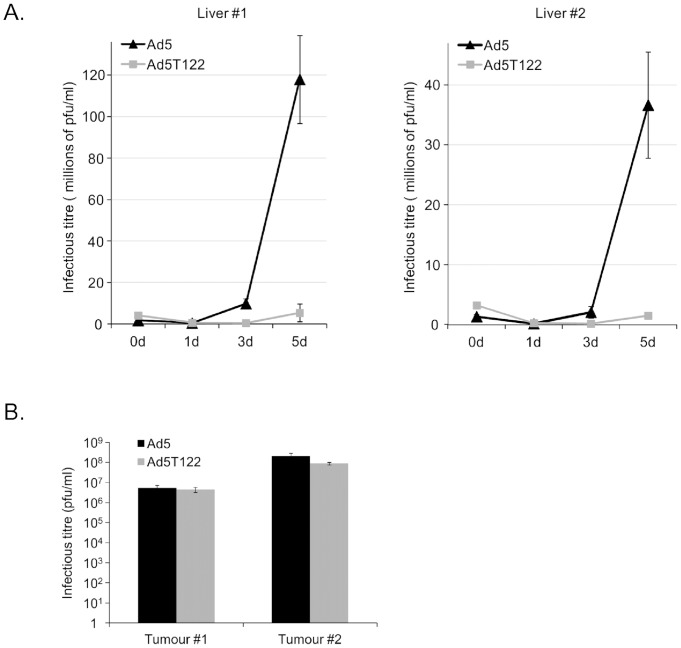
Quantitative analysis of productive replication of Ad5 and Ad5T122 in human liver and in liver metastatic colorectal cancer tissue. (**A**) Titration of infectious virus in the supernatants of liver tissue cultures collected 1 h (0d), 1d, 3d, or 5d after infection with 10^7^ PFU of Ad5 or Ad5T122. (**B**) Infectious titre in supernatants of the tumour tissue infected with 10^7^ PFU of Ad5 or Ad5T122 were quantitated 5 d after the infection. The virus titres are indicated as the tissue culture infective dose 50 (TCID50) on the y-axis. The data are presented as the mean of triplicates ± standard error.

Immunohistochemistry of the infected tissues does not provide a quantitative measure of viral replication, and low levels of E1A do not exclude replication of the miR122-targeted virus. Therefore, to directly examine the rate of productive replication of Ad5 and Ad5T122 in the human liver and colorectal cancer liver metastasis tissues the amount of infectious virus in the medium of these *ex vivo* cultures was quantitated at different time point after the virus inoculum. Data from titration experiments done in triplicate to determine the tissue culture infective dose 50 (TCID_50_) are shown in Figure 4. To minimize the manipulation of these tissues the input virus (10^7^ PFU/ml) was not removed, which thus accounted for the infectious titre measured 1 h p.i. (0 d time point in Figs. 4A and 4B).

The infectious titre of Ad5 in the liver tissue supernatants increased robustly during the follow-up period, exceeding the input dose by 68-fold at the 5d time point for liver #1 and by 27-fold for liver #2. By contrast, very little infectious Ad5T122 was found in the liver tissue supernatants, where the titre for liver #1 only slightly (1.3-fold) exceeded the input (1 h p.i.) level at the final 5d time point, and for liver #2 even decreased to less than half (0.47-fold) of the original input. In samples of colorectal cancer liver metastasis tissue both viruses replicated to the same extent (Fig. 4B). Based on these data we conclude that the miR122 sites could strongly suppress replication of Ad5T122 in normal human liver tissue without compromising its replication in colorectal cancer liver metastasis tissue.

### Systemic Injection of Ad5 but not Ad5T122 Leads to Hepatic Damage in Mice

**Figure 5:**
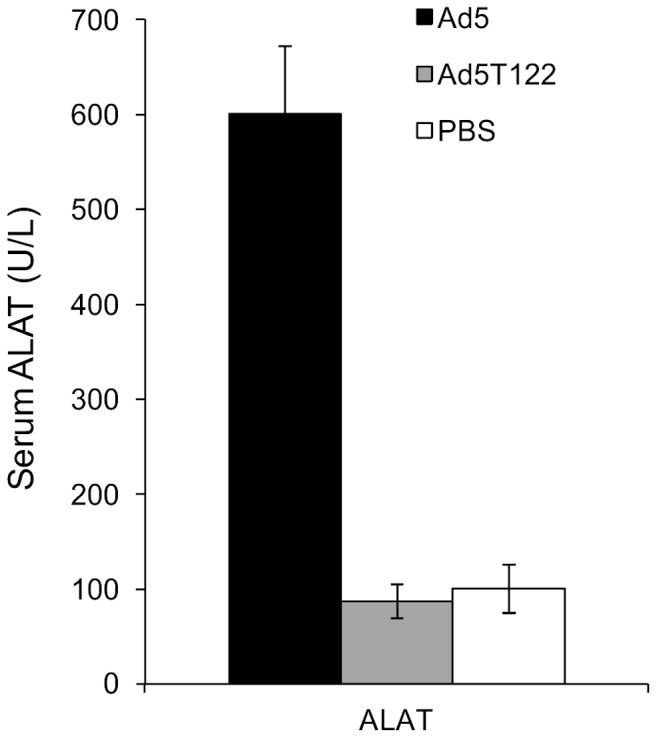
Assessment of hepatotoxicity of Ad5 and Ad5T122 systemically administered into mice. Measurement of serum ALAT levels 72 h after systemic injection of 10^9^ PFU of Ad5, Ad5T122 or PBS (Mock). Data are presented as mean ± standard error. Four animals per group were used.

Despite the failure of human adenovirus to productively replicate in murine cells systemic viral injection is associated with signs of hepatocyte damage, such as elevated serum levels of the liver enzyme alanine aminotransferase (ALAT). To investigate the behaviour of Ad5T122 in this mouse model of adenovirus-induced liver toxicity we injected 1×10^9^ PFU of Ad5, Ad5T122 or PBS into the tail vein of C57bl/6 mice. 72 h post-injection these animals were sacrificed and serum ALAT levels were measured. Mice injected with Ad5 showed significantly increased ALAT levels compared to PBS injected control mice, indicating that hepatocyte damage had indeed occurred. By contrast, mice injected with Ad5T122 showed ALAT levels that were indistinguishable from those of PBS injected mice (Fig. 5).

### Ad5T122 and Ad5 have Similar Oncolytic Potential in a Lung Cancer Xenograft Model

**Figure 6:**
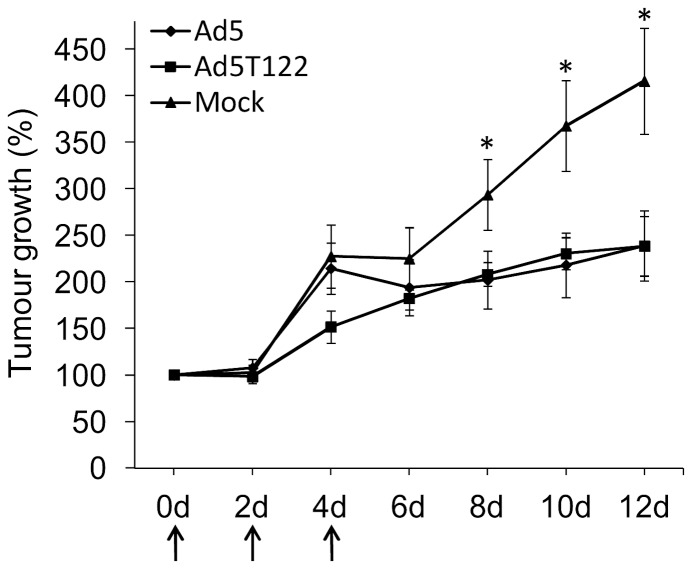
Oncolytic potential of Ad5T122 and Ad5 in a lung cancer xenograft model. Mice were treated with 10^7^ PFU of Ad5, Ad5T122, or injected with a corresponding volume of PBS (Mock) on days 0, 2 and 4, as indicated by arrows. Tumour size was calculated as the volume of an ellipsoid. Tumour growth is expressed as a percentage increase from first day of virus injection. The asterisks (*) indicate a statistically significant differences in the volumes of the PBS-injected tumours compared to the virus-treated tumours. The number of tumours in each group were 10 (PBS), 11 (Ad5T122), and 12 (Ad5). Data are presented as mean ± standard error.

Although Ad5T122 replicated well and was as cythopathic as Ad5 in non-hepatic tumour cell lines (Figure 2) it was important to confirm that its attenuation in liver was not associated with a reduced oncolytic potential in a more relevant model of cancer virotherapy. Therefore, groups of nude mice bearing an established subcutaneous A549 lung cancer xenografts with initial volumes typically ranging between 15–40 mm^3^ were treated intratumourally on days 0, 2 and 4 with 1×10^7^ PFU of Ad5, Ad5T122 or a corresponding volume of PBS, and tumour growth was monitored for 12 days following the start of the treatment. The group of mice receiving PBS showed rapidly increasing tumour growth, and by day 12 after the start of the treatment the average volume of the tumours had reached more than 5× the initial volume. The groups of mice receiving either Ad5 or Ad5T122 showed very similar oncolytic potential and significantly reduced tumour growth from day 8 compared to the PBS treated group (Fig. 6). Based on these data we conclude that the oncolytic potency of Ad5T122 was not reduced compared the wild-type Ad5.

## Discussion

In this study we show that targeting of E1A to cell type-specific downregulation by miR122 is sufficient to potently attenuate adenovirus replication in the human liver. These observations emphasize the utility of miRNA-mediated optimization of the tropism of virotherapy in general, and its use in liver detargeting of oncolytic adenoviruses in particular.

Using a chimeric Ad5/3 adenovirus containing three miR122 target elements in the E1A 3′UTR, we have previously reported that despite potent suppression of E1A mRNA and protein expression, viral replication was only modestly attenuated in the liver-derived Huh7 cells [22]. In order to bring the replication of this modified Ad5/3 virus effectively under the control of miR122-mediated E1A regulation, it was necessary to combine the liver-specific, miR122-mediated inhibition with a mutation that non-specifically decreased E1A translation in all cell types. By itself this uniform decrease of E1A protein production did not have a noticeable effect on Ad5/3 replication in a panel of non-hepatic cancer cell lines, but together with the miR122-mediated E1A control led to a potent suppression of replication and cytopathicity in Huh7 cells [22].

Subsequently Cawood et al. reported the use of a serotype 5 virus in which E1A had been replaced with an E1A-luciferase fusion gene containing four miR122 target elements in its 3′UTR. This chimeric mRNA was strongly downregulated by miR122, as evidenced by reduced luciferase activity in Huh7 cells and in primary hepatocyte cultures, as well as in the livers of infected mice. They also showed that serum markers of liver damage as well as viral genome copy numbers in the livers of mice infected with wild-type Ad5 containing four miR122 targets were lower than mice infected with an unmodified virus [23], [24]. While promising, it is difficult to extrapolate these findings to the infection of human liver, since human adenoviruses are not able to productively replicate in mouse cells [30]. Nevertheless, it is interesting to note that despite this limited capacity to replicate in murine cells, our current findings together with the data by Cawood et al. clearly show that the elevated markers of liver damage in mice are a genuine consequence of adenoviral gene expression in hepatocytes rather than due to less direct hepatotoxicity caused by the viral particles, and can thus be counteracted by miR122-based targeting.

Cawood et al. suggested that four miR122 target sites as compared to the three copies used in our previous study allowed a better control of viral replication. However, no data was provided on infection of Huh7 or other liver-derived cells that would be competent for adenovirus replication. On the other hand, similar to our earlier study, Leja et al. reported that combining miR122-mediated E1A mRNA suppression with other inhibitory measures was required to potently suppress adenovirus replication in cultured hepatic cells [25]. Specifically, they combined miR122-mediated downregulation of E1A with deletion of the E1B gene and a tissue-specific promoter showing low activity in the liver to drive E1A transcription.

The potent suppression of the current Ad5T122 virus in the normal human liver tissue may be contributed by the higher number of miR122 targets compared to the Ad5/3-derived virus that we have studied earlier [22] (six vs. three copies). However, Leja et al. also used six miR122 targets in their related study design discussed above. Moreover, while Ad5T122 was clearly more attenuated in Huh7 cells than our previous Ad5/3-derivative [22], residual replication/cytopathicity was still observed in the Ad5T122-infected Huh7 cells (see Figure 2). Thus, the profound attenuation of Ad5T122 observed in the *ex vivo* cultured human liver (Figure 4A) was likely related to the non-transformed phenotype and the normal tissue environment of the hepatocytes in the *ex vivo* tissue culture. Indeed, E1A provides several key functions for adenovirus replication, which are differentially needed in normal vs. transformed cells [33]. Interactions of E1A with host cell proteins, such as the tumour suppressor Rb, modulate the activity of transcription factors and cell-cycle regulators, thereby promoting further viral gene expression and pushing the host into the S-phase of cell cycle to enable viral DNA replication. Since these signalling pathways are inherently deregulated in transformed cells [34], such as Huh7, adenovirus replication may proceed normally even if the E1A levels are greatly reduced [22], [35]. On the other hand, although the rate of proliferation of primary hepatocytes grown on cell culture dishes depend on many factors, such culturing effectively promotes the transition of their cell cycle towards the S-phase [36], [37]. Thus, although representing a more physiological study system than the Huh7 hepatocarcinoma cell line, cultured primary hepatocytes do not provide a good model for addressing the effects of suppressed E1A expression on adenovirus replication in the liver. Finally, it is also important to note that Huh7 cells express only 8% of the miR122 levels observed in primary human hepatocytes [27].

Indeed, miR122 is very highly expressed in normal hepatocytes where it has been estimated to constitute over 70% of all miRNAs expressed [27], [38]. Equally important, miRNA122 is one the most tissue-specific miRNA and apart from minimal expression in some cells of the thymus and brain, it is not expressed outside of the liver [38], [39], [40], [41].

Although even a strong systemic antagomir-mediated miR122 inhibition used as an experimental HCV therapy did not show any obvious adverse effects on the liver [42], the prominent role of miR122 in hepatocytes might raise concerns of disrupting normal gene regulation in hepatocytes due to a “sponge effect” of expression of multiple miR122 target sites. However, this is not a relevant concern in our approach because of a strong inbuilt negative feedback loop in the Ad5T122 virus. The number of miR122 target elements is determined by the copy number of E1A mRNAs in the infected cells, which becomes very low in liver cells due to miR122-guided destruction. Moreover, the failure of Ad5T122 to replicate and spread in the liver cells provides another layer of protection against deregulating normal miR122-regulated processes in the liver.

The success in using miR122 targets for suppressing E1A expression, suggests that similar strategies might also be useful for preventing potentially harmful replication of other therapeutic or vaccine viruses. Moreover, miR122-control could also be further employed in liver detargeting of oncolytic adenoviruses by placing additional target sequences in other positions in the viral genome. Compared to E1A, targeting of an mRNA encoding for a structural viral protein might result in a potent attenuation even if a less complete shut-off of the target gene expression would be achieved. Conversely, however, maximal replication in tumour cells might be compromised even by minor reduction in the levels of the modified mRNA, which is not a concern in the case of E1A.

It has been reported that viral sequestration into the liver and subsequent infection of the hepatocytes following systemic administration of serotype 5 adenoviruses can be reduced by introducing mutations into the Ad5 hexon protein that abolish binding of the viral capsid to the blood coagulation factor X (FX) [43], [44]. Combining such transductional liver-detargeting with the post-transcriptional, miR122-based approach validated in this study would be straight-forward, and could further minimize the potential damage to hepatocytes by oncolytic adenoviruses, especially when treating tumours outside of the liver.

Of note, when considering intrahepatic tumours, the value of miR122-based targeting is not limited to metastatic disease, but could also be exploited in virotherapy of primary hepatocellular carcinoma (HCC). Reflecting its role as a tumour suppressor [45], [46], the loss of miR122 expression is common in HCC [47], [48], and the Huh7 cells used in this study are exceptional among HCC-derived cell lines in resembling normal hepatocytes by expressing miR122 [27].

It seems likely that the future oncolytic adenoviruses to be used in the clinics will be “armed viruses” that express at least one additional gene product aimed at enhancing direct or immune-mediated killing of the infected cells, and promoting the development of systemic anti-tumour immunity [14], [49]. Preventing the expression of such potentially dangerous effector genes in hepatocytes might be even more critical than suppressing viral replication itself. Thus, placing these toxic or immunomodulatory genes under a dual miR122 control by targeting their mRNAs directly as well as indirectly (via miR122-regulated replication) would provide a synergistic and powerful strategy for excluding their expression in hepatocytes.

In summary, the current study provides a definitive proof of concept and preclinical validation for the use of miR122 target elements for reducing the risk of liver toxicity of therapeutic adenoviruses. Our data show that in normal human liver tissue miR122 target elements alone are sufficient to profoundly attenuate Ad5. However, due to the ease of introduction and the small genomic size of this modification, it should be possible and beneficial to combine it with any other adenoviral targeting approach.

## Materials and Methods

### Ethics Statement

Human tissue specimens were obtained with written informed consent and approval by ethics committee of the Helsinki University central hospital. All animal experiments were approved by the experimental animal committee of the University of Helsinki and the provincial government of Southern Finland.

### Cell Lines

Human lung carcinoma cell line A549, human colorectal cancer cell line HCT116 and human epidermoid carcinoma cell line HEp-2 was obtained from ATCC (Manassas, VA). Human embryonic kidney cell line 293 was purchased from Microbix (Toronto, Canada). Human hepatocellular carcinoma cell line Huh7 [50] was a gift from Professor Mark Harris (Leeds University, UK). All cell lines were cultured in DMEM with 10% foetal calf serum (FBS) (Life Technologies) 1% L-glutamine and 1% penicillin/streptomycin at 37°C/5% CO_2_.

### Dual-luciferase Assays

Cells were transfected with Renilla- and firefly-luciferase plasmids as described previously [22] and 48 h post-transfection luciferase activities were measured using Dual-Luciferase Assay System (Promega).

### Construction and Production of Adenoviruses

pShuttle 6×122 was made as described [22], except inserting six copies of miR122 target elements instead of three. Homologous recombination between the modified subgenomic adenoviral genome and pAd5-Δ24 [51], and generation of infectious adenovirus stocks were done using previously described standard procedures [22]. The titres of the purified viruses were: 2.12×10^12^ VP/ml and 1.12×10^11^ PFU/ml for Ad5, 1.28×10^12^ VP/ml and 3.98×10^10^ PFU/ml for Ad5T122 and 1.40×10^12^ VP/ml and 4.47×10^10^ PFU/ml for Ad5Luc1. All VP/PFU ratios were below 35 indicating good quality of the virus preparations. Intact of miRNA target elements were verified also by sequencing analyses of the purified viral stocks.

### Virus Replication and Cell Viability Assays

Viruses were titrated by using tissue culture infective dose 50 (TCID_50_). Briefly, permissive 293 cells were seeded in 96-well plates (10^4^ cells/well) and the next day eight serial 10-fold dilutions of purified viruses or collected supernatants were subjected to the 96-well plate. After 10 days of incubation the wells with observable CPE were counted, and the viral titres calculated calculated by the Kärber-Spearman method [52]. Cell viability was measured using the CellTiter 96 AQ_ueous_ One Solution Cell Proliferation Assay (Promega, Fitchburg, WI), and a multi-well plate reader (Multiskan EX; Thermo Fisher Scientific, Waltham, MA) to determine the optical density of the reactions at 490 nm.

### Argonaute 2 Knockdown Cell Lines

pLKO.1-puro-CMV-TurboGFP and two different pLKO.1-puro-shRNA-Ago2 (The RNAi Consortium (TRC) ID numbers: TRCN0000007865 and TRCN0000007866) expression plasmids were transfected into 293T cells together with packaging plasmids to generate lentiviral preparations using standard procedures. Transduced Huh7 cells stably expressing shRNA against Argonaute 2 (Ago2) or a control lentiviral genome (TurboGFP) were obtained by a selection with 2.5 µg/ml puromycin for three days.

### miR122 Inhibitor Assay

Huh7 cells were transfected in 12-well plates with 200 nM miRIDIAN miR122 inhibitor (Dharmacon) or 1.6 µg of salmon sperm DNA as a control using Lipofectamine 2000 (Invitrogen) and 24 h post-transfection cells were infected with 400 000 PFU of Ad5 or Ad5T122. Cells were photographed six days later to document the appearance of a cytopathic effect.

### *Ex vivo* Tissue Culture and Infections

Healthy human liver or a liver metastasis of colorectal carcinoma were obtained with written informed consent and approval by ethics committee of the Helsinki University central hospital. A cylinder of 8 mm in diameter was cored out of the surgically removed tissue and sliced at approximately 300–500 µm with a Krumdieck precision-cut tissue slicer (Alabama Research and Development Corporation, Huntsville, AL). Tissue slices were placed into six-well plates (1 slice/well) containing 2 ml of William’s Medium E with 10% foetal calf serum (FBS) (Life Technologies), 1% Glutamax-I (Life Technologies) and 1% penicillin/streptomycin. The plates were incubated at 37°C/5% CO_2_ in a humidified environment for up to 5 days. A plate rocker was used to gently agitate the slices to ensure adequate oxygenation and viability. Slices were infected by adding 2×10^6^ or 10^7^ PFU of virus into the medium.

### Immunohistochemistry (IHC)

Slices were fixed o/n in 10% phosphate-buffered formalin, dehydrated, embedded in paraffin and cut into 4 µm sections. The sections were deparaffinised, rehydrated and either stained with haematoxylin and eosin, or prepared for immune staining by heating the slides in Tris–EDTA (pH 9.0) buffer in a microwave oven. Antibody against Adenovirus 5 E1A (MS 1069-P0, Neomarkers, Thermo Fisher Scientific, 1∶200 dilution) was used as a primary antibody. Dako Envision system (Dako, Agilent Technologies, Santa Clara, CA) including HRP-conjugated anti-mouse secondary antibody was used for detection. Labvision autostainer (Thermo Fisher Scientific) was used for IHC sample preparation.

### In vivo Experiments for Assessment of Liver Toxicity

Adult 5 to 7 week old female C57bl/6 mice were systemically infected with 1×10^9^ PFU of Ad5 or Ad5T122 by tail vein injection (diluted in 100 µl of PBS). Control group received 100 µl of PBS. 4 animals were used in each group. After 72 h post-infection blood was taken from mice by cardiac puncture and allowed to clot for 15 min at room temperature and spun at 1200 g for 10 min. Serum samples were added to ALAT reagent (Thermo Fisher Scientific) and the change in absorbance (340 nm) per minute was monitored. Units of ALAT activity were calculated according to manufacturer’s instructions.

### In vivo Experiments for Assessment of Oncolytic Potency

Adult 5 to 7 week old female NMRI nude mice were subcutaneously injected with 3×10^6^ A549 cells into both flanks. Initial tumour sizes were typically 15–40 mm^3^. Mice were treated on days 0, 2 and 4 by intratumoural injection at 10^7^ PFU/tumour with either Ad5 or Ad5T122 (diluted in 50 µl of PBS). Control group received 50 µl of PBS. 6 animals were used in each group. In order to determine tumour volume by hand-held calliper, the greatest longitudinal diameter (length) and the greatest transverse diameter (width) were determined. Tumour volumes were calculated by the modified ellipsoidal formula [53], [54]: Tumour volume = 0.5(length×width^2^). MedCalc software was used to calculate serial measurements (area under curve) for statistical analysis. A P value of less than 0.05 was considered as significant.

## Supporting Information

Figure S1 Functional quantitation of miR122 expression in different cell lines. The indicated cells lines were co-transfected with an unmodified Firefly luciferase vector (pSIRNALUC-3′MluI) or its derivative containing a miR122 target element in the 3′ UTR (pSIRNALUC-3′1×T122) together with a vector for Renilla luciferase (pcDNA-Renilla). The average Firefly/Renilla luciferase activity ratios in these cells 48 hours after transfection is shown on the y-axis. The value from pSIRNALUC-3′MluI -transfected cells was set to 100%, and the ratio from pSIRNALUC-3′1×T122 -transfected cells is expressed relative to this. The data are presented as the mean of triplicates ± standard error.
(TIF)

Figure S2 Suppression of Ad5T122 replication in Huh7 cells is miR122-specific. **A.** Effect of miRNA machinery disruption by down-regulation of Argonaute 2 on Ad5T122 replication in Huh7 cells. Two independent Huh7 cell lines (Ago2-kd1 and Ago-kd2) stably expressing different shRNA constructs targeting Ago2 or a control Huh7 derivative cell line (Control) transduced with a control lentiviral vector were infected with 400 000 pfu of Ad5 or Ad5T122, or left uninfected as indicated, and photographed 6 days post-infection. **B.** Effect of miR122 inhibition by a synthetic antagomir oligonucleotide on Ad5T122 replication in Huh7 cells. Cells were transfected with the miR122 inhibitor or mock transfected, infected with 400 000 PFU of Ad5 or Ad5T122 on the next day, and photographed 6 days post-infection.
(TIF)
