# Supplementary figures and images for: MicroRNA-Mediated Suppression of Oncolytic Adenovirus Replication in Human Liver
Source: PLoS One. 2013 Jan 22;8(1):e54506. doi: 10.1371/journal.pone.0054506 (PMC3551754; doi:10.1371/journal.pone.0054506)

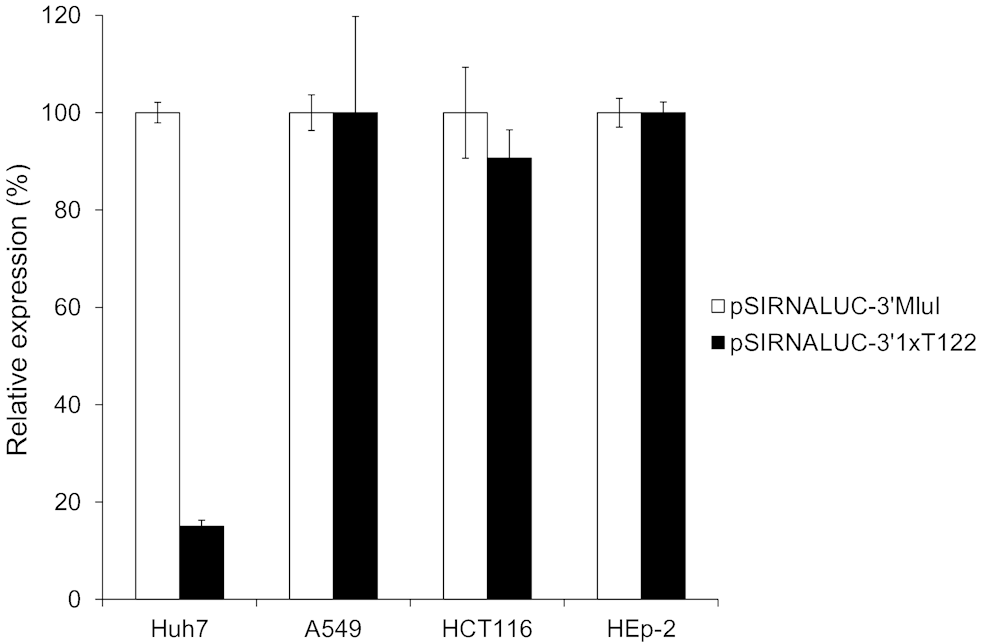

Supplement: Figure S1 — Functional quantitation of miR122 expression in different cell lines. The indicated cells lines were co-transfected with an unmodified Firefly luciferase vector (pSIRNALUC-3′MluI) or its derivative containing a miR122 target element in the 3′ UTR (pSIRNALUC-3′1×T122) together with a vector for Renilla luciferase (pcDNA-Renilla). The average Firefly/Renilla luciferase activity ratios in these cells 48 hours after transfection is shown on the y-axis. The value from pSIRNALUC-3′MluI -transfected cells was set to 100%, and the ratio from pSIRNALUC-3′1×T122 -transfected cells is expressed relative to this. The data are presented as the mean of triplicates ± standard error. (TIF) [file pone.0054506.s001.tif]

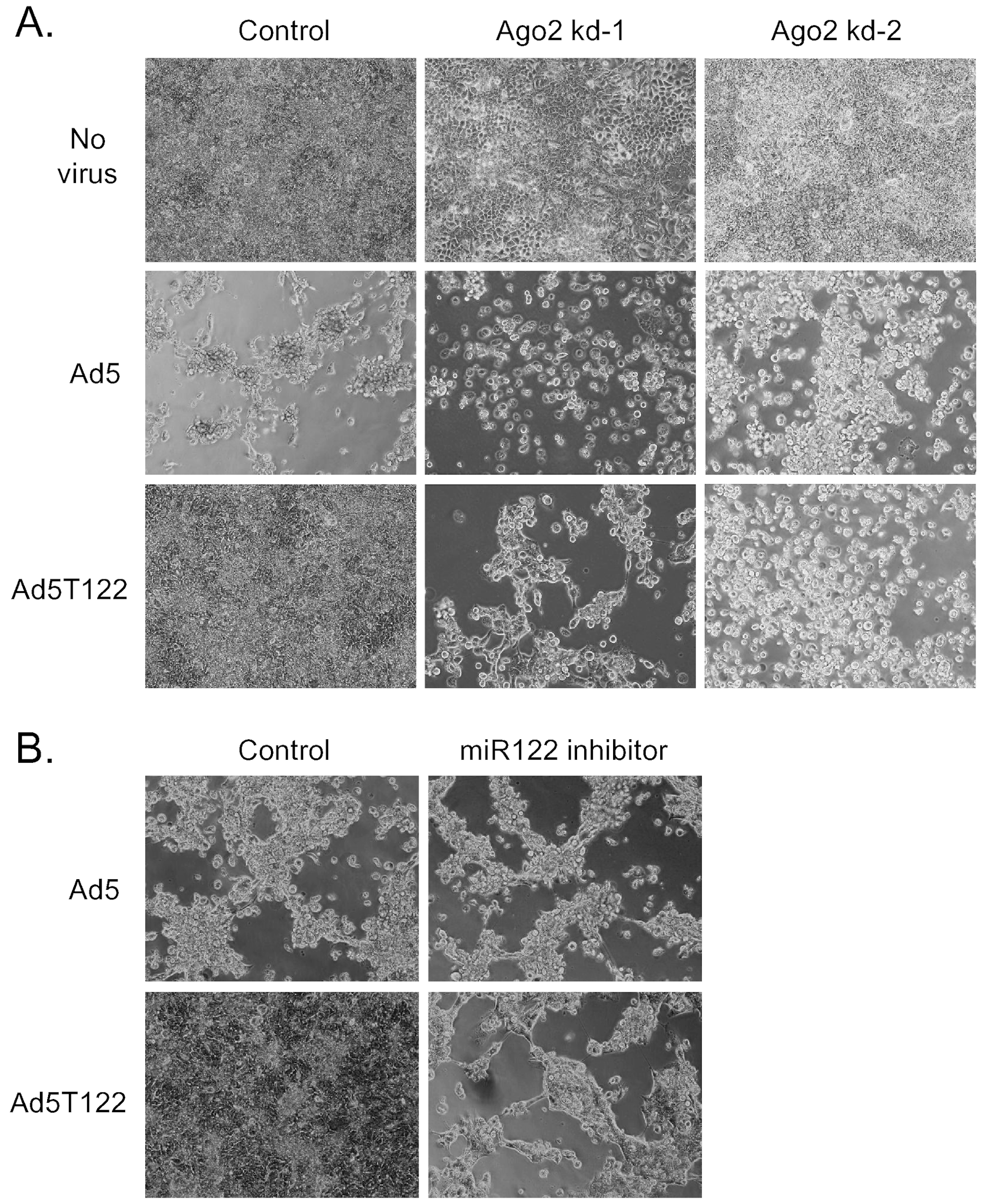

Supplement: Figure S2 — Suppression of Ad5T122 replication in Huh7 cells is miR122-specific. A. Effect of miRNA machinery disruption by down-regulation of Argonaute 2 on Ad5T122 replication in Huh7 cells. Two independent Huh7 cell lines (Ago2-kd1 and Ago-kd2) stably expressing different shRNA constructs targeting Ago2 or a control Huh7 derivative cell line (Control) transduced with a control lentiviral vector were infected with 400 000 pfu of Ad5 or Ad5T122, or left uninfected as indicated, and photographed 6 days post-infection. B. Effect of miR122 inhibition by a synthetic antagomir oligonucleotide on Ad5T122 replication in Huh7 cells. Cells were transfected with the miR122 inhibitor or mock transfected, infected with 400 000 PFU of Ad5 or Ad5T122 on the next day, and photographed 6 days post-infection. (TIF) [file pone.0054506.s002.tif]
